# An evaluation of injurious falls and Fall-Risk-Increasing-Drug (FRID) prescribing in ambulatory care in older adults

**DOI:** 10.1186/s12877-022-02877-z

**Published:** 2022-03-10

**Authors:** Taylor R. Elliott, Susan Westneat, Shama D. Karanth, Erin L. Abner, Anna M. Kucharska-Newton, Daniela C. Moga

**Affiliations:** 1 University of Kentucky College of Pharmacy, Lexington, KY 40356 USA; 2 University of Kentucky College of Public Health, Lexington, KY 40356 USA; 3 Durham Veterans Affairs Medical Center, Durham, NC USA; 4 Institute On Aging, University of Florida, Gainesville, FL USA; 5 Sanders-Brown Center On Aging, Lexington, KY 40356 USA; 6 The Gillings School of Global Public Health, University of North Carolina at Chapel Hill, Chapel Hill, NC USA

**Keywords:** Fall-risk-increasing-drugs (FRIDs), Injurious falls, Older adults, Prescribing behaviors

## Abstract

**Background:**

Falls are a major public health problem affecting millions of older adults each year. Little is known about FRID prescribing behaviors after injurious falls occur. The primary objective of this study was to investigate whether an injurious fall is associated with being prescribed a new FRID.

**Methods:**

We conducted a cross-sectional analysis using data from the National Ambulatory Medical Care Survey (2016). We included visits from patients age ≥ 65 years and classified visits based on presence of an injurious fall. The outcome of interest was prescription of new FRID between those with and without an injurious fall. Multivariable logistic regression weighted for sampling and adjusted for demographics, health history and other medications was used. Age and Alzheimer’s disease were examined as potential effect measure modifiers. Odds ratios and 95% confidence intervals were reported. Bayes factor upper bounds were also reported to quantify whether the data were better predicted by the null hypothesis or the alternative hypothesis.

**Results:**

The sample included 239,016,482 ambulatory care visits. 5,095,734 (2.1%) of the visits were related to an injurious fall. An injurious fall was associated with a non-statistically significant increase in odds of at least one new FRID prescription: adjusted OR = 1.6 (95% CI 0.6, 4.0). However, there was non-statistically significant evidence that the association depended on patient age, with OR = 2.6 (95% CI 0.9, 7.4) for ages 65–74 versus OR = 0.4 (95% CI 0.1, 1.6) for ages ≥ 75. In addition to age, Alzheimer’s disease was also identified as a statistically significant effect measure modifier, but stratum specific estimates were not determined due to small sample sizes.

**Conclusions:**

Ambulatory care visits involving an injurious fall showed a non-statistically significant increase in odds of generating a new FRID prescription, but this association may depend on age.

**Supplementary Information:**

The online version contains supplementary material available at 10.1186/s12877-022-02877-z.

## Background

Falls are a major public health problem that affects millions of people in the United States each year. Older adults (specifically those 65 years of age and older) are disproportionately affected, with one out of four older adults experiencing a fall each year according to data from the Centers for Disease Control and Prevention [1]. Approximately 20% of these falls result in a serious injury, most often a fractured or broken bone or a traumatic brain injury (TBI) [1]. Injurious falls initiate a cascading effect that can negatively impact the patient’s physical and mental health. For example, patients experiencing hip fractures often require orthopedic surgery, which puts them at increased short-term risk for infection and bleeding, as well as increased long-term risk for deep venous thrombosis (DVT) and muscle atrophy due to prolonged immobility [2]. In addition, patients often have a fear of falling a second time, which can cause them to be less mobile, therefore compounding the probability of a subsequent fall [1, 3, 4]. The economic costs associated with injurious falls place an enormous burden on the healthcare system. Each year, approximately $50 billion and $754 million is spent on non-fatal and fatal fall injuries, respectively [5].

Numerous risk factors for falls have been elucidated, and they are generally subclassified as extrinsic or intrinsic. Extrinsic factors are related to the physical environment (i.e., dim lighting, improper use of assistive devices, etc.) and are often modifiable. Intrinsic factors are related to patient-specific factors, such as advanced age, history of previous falls, chronic conditions, and the physiologic effects of medication use [1, 5–8]. More specifically, medications belonging to certain medication classes can independently increase fall risk and are known as fall-risk-increasing drugs (FRIDs).

The CDC implemented a program called Stopping Elderly Accidents, Deaths, & Injuries (STEADI)-Rx that is designed to help pharmacists identify medications of concern [9]. The American Geriatrics Society (AGS) 2019 Updated AGS Beers Criteria for Potentially Inappropriate Medication Use in Older Adults (hereafter “Beers Criteria”) created a similar list of medications and medication classes to avoid in patients with a history of falls or fractures [10]. These medication classes include anticonvulsants, antidepressants, antihypertensives, antipsychotics, antispasmodics, benzodiazepines, opioids, and sedative hypnotics [11–13]. Polypharmacy, which is generally defined as a medication regimen consisting of five or more medications, is also associated with increased fall risk, with one study estimating a 7% increase in fall risk with every additional medication reported [14].

While FRID classes have been elucidated, little is known about FRID prescribing behaviors after injurious falls occur. The primary objective of this study was to examine prescribing behaviors occurring during a visit for an injurious fall and to investigate whether an injurious fall is associated with being prescribed a new FRID. The secondary objective of this study is to describe the pharmacist’s role in fall prevention. Analyses described herein are based on prescribing behaviors ascertained from the 2016 National Ambulatory Medical Care Survey (NAMCS), which was the most recently available data at the time data analysis was conducted and finalized in April 2021.

## Methods

### Study population and data source

NAMCS is a nationally representative cross-sectional survey that provides information about the utilization of ambulatory medical care services in the United States. Each participating office-based physician is assigned to a 1-week reporting period randomly, and a systematic random sample of visits is recorded during that timeframe using a computerized form [15]. Data provided by participating physicians include patient characteristics such as demographic information; diagnoses and chronic conditions (including total number of chronic conditions); services ordered or provided; laboratory values; and treatments, including up to 30 medication therapies. NAMCS is a publicly available, deidentified data source. The surveys are administered by the National Center for Health Statistics that is part of the Centers for Disease Control and Prevention (CDC) in the US. The data collected are made publicly available by the CDC along with sampling weights and guidance in applying the weights to obtain nationally representative estimates. A detailed description of NAMCS sampling methodology is available from the National Center for Health Statistics [16]. The study was exempt from Institutional Review Board approval at the University of Kentucky. All methods were performed in accordance with the relevant guidelines and regulations.

### Eligibility criteria

For this study, we included visits for patients age 65 years or older, as available lists of potentially inappropriate medications (PIMs) are typically based on this age criterion [9, 10]. Adults less than 65 years of age were excluded.

### Identification of injurious falls

**Fig. 1:**
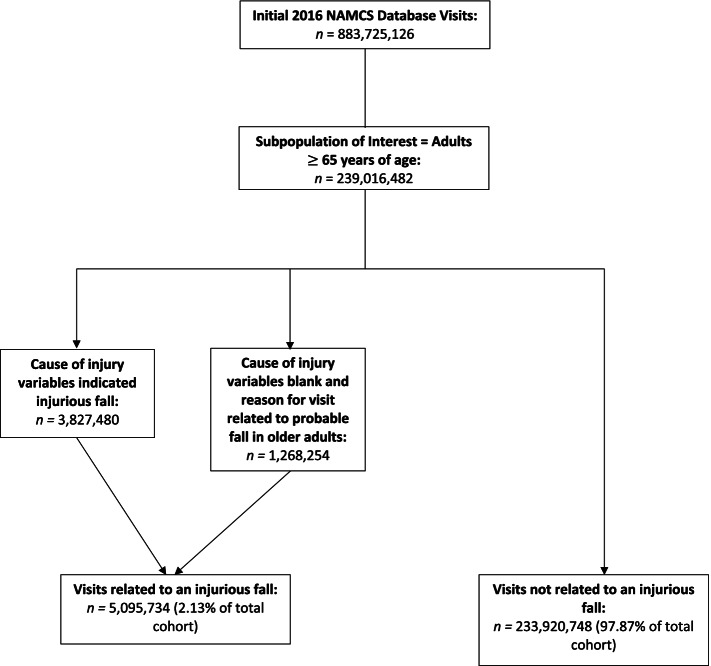
Cohort Identification (weighted sample)

We created an indicator to identify the presence of injurious falls (Fig. 1). Under the “Injury” section on the NAMCS standard form, the physician selected whether the visit was related to an injury/trauma, overdose/poisoning, or adverse effect of medical/surgical treatment. For visits related to injury/trauma, we then manually selected each injury caused by a fall from the free-text fields for the cause1, cause2, and cause3 variables. We also investigated the “Reason for Visit” section on the NAMCS standard form when the causes of injury were left blank. We identified reasons for visits related to probable falls in older adults (e.g., fracture/dislocation of arm or wrist). Please see additional file 1: Appendix 1 for the values corresponding to injurious falls identified in the cause of injury variables (CAUSE1, CAUSE2, and CAUSE3), as well as the reason for visit variables (RFV1, RFV2, RFV3, RFV4, and RFV5).

### Identification of FRIDs and Polypharmacy

The survey data collection form allows up to 30 medications to be reported. The physician indicates whether the medication was new or continued at the time of the visit. The medications are then assigned to up to four therapeutic classes using the Lexicon Plus® database and the Multum classification system [17]. Based on the lists provided by STEADI-Rx and the Beers Criteria, we created new, continued, and new or continued indicator variables for each medication class of concern: anticonvulsants, antidepressants, antihypertensives, antipsychotics, antispasmodics, benzodiazepines, opioids, and sedative hypnotics [9, 10]. New medications were those prescribed at the visit, and continued medications were those already part of the patient’s medication regimen prior to the visit. See additional file 1: Appendix 2 for the Multum therapeutic class codes corresponding to the medication classes of concern.

In addition to the indicator variables for each medication class of concern, we also created an indicator variable to show whether any new, continued, or new or continued FRID belonging to any FRID class listed above was present. We also created continuous variables to indicate the total number of new, continued, and new or continued FRIDs present.

Lastly, we created an indicator variable to identify the presence of poly-FRID. Poly-FRID is defined as a medication regimen consisting of two or more FRIDs and has been shown to be an independent risk factor for falls [18]. For the poly-FRID variable, we only considered continued medications in order to assess medication use prior to the visit.

### Covariates (potential confounders)

**Fig. 2:**
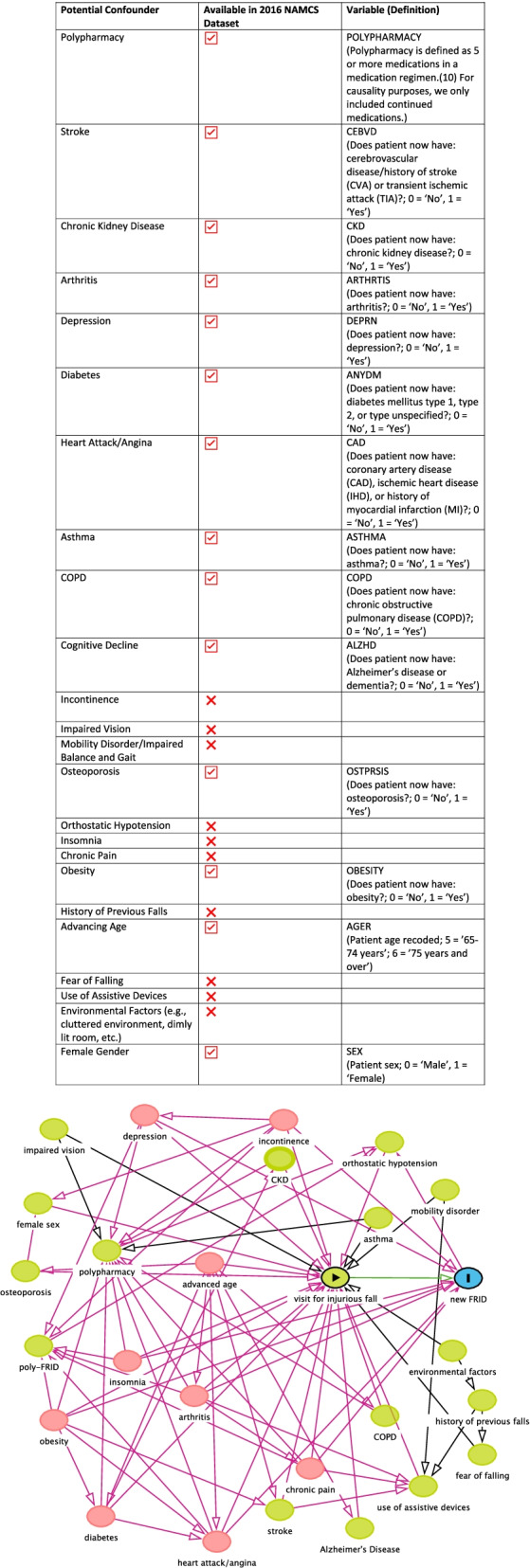
Potential Confounders Minimal sufficient adjustment set for estimating the total effect of visit for injurious fall on new FRID: Alzheimer’s disease, CKD, COPD, arthritis, asthma, chronic pain, depression, diabetes, heart attack/angina, impaired vision, incontinence, insomnia, obesity, orthostatic hypotension, polypharmacy, stroke. *Chronic pain, impaired vision, incontinence, insomnia, and orthostatic hypotension not available, **CKD: chronic kidney disease; COPD: chronic obstructive pulmonary disease

We conducted a literature review to identify important risk factors for falls [8, 19–21]. Figure 2 lists potential confounders (i.e., shared causes of falls and medication use), whether or not they were available in the NAMCS data, and how they were defined if included in the analyses. We included Alzheimer’s disease, chronic kidney disease (CKD), chronic obstructive pulmonary disease (COPD), arthritis, asthma, depression, diabetes mellitus, heart attack/angina, obesity, polypharmacy, and stroke in the multivariable logistic model, based on the minimally sufficient adjustment set identified by the hypothesized causal diagram. Chronic pain, impaired vision, incontinence, insomnia, and orthostatic hypotension were also part of the minimally sufficient adjustment set but were not available from the NAMCS data.

### Data Analyses

We applied NAMCS sampling weights to account for complex sampling design and to generate a nationally representative sample of ambulatory care visits for patients 65 years of age or older [16]. Proportions were reported for nominal variables of interest. Continuous variables of interest were described using the mean and standard error (SE). Chi-squared tests were used to compare characteristics between the injurious fall group and the non-fall group, as appropriate. To assess whether new FRID prescriptions were more or less likely between the two groups, multivariable logistic regression was used. Age (65–74 years versus 75 years and older, per NAMCS coding) was examined as a potential effect measure modifier, given the dramatic increase in fall-related mortality rate in persons 75 years of age and older [22]. Adjusted odds ratios (OR) and 95% confidence intervals (95% CI) were reported, with *p*< 0.05 considered statistically significant. Bayes factor upper bounds were reported to quantify whether the data were better predicted by the null hypothesis or the alternative hypothesis [23]. Alzheimer’s disease was examined as a potential effect measure modifier, as certain medications (i.e., psychotropics with anticholinergic properties) can both exacerbate cognitive impairment and increase the risk for subsequent falls. However, no model estimates are presented due to sparse data [24, 25].

## Results

### Baseline demographics

A total of 239,016,482 ambulatory care visits occurring among patients 65 years or older were included in the analyses (Table 1). Of those, 5,095,734 (2.1%) visits were related to an injurious fall. The mean (SE) age for the 239,016,482 adults 65 years of age or older represented by study visits was 74.8 (SE 0.2). Non-Hispanic white patients made up 78.1% of the visits. The majority of visits were made by women (55.9%). Prevalence of chronic conditions recorded at the time of the visit was similar between those with and without injurious falls (mean of 2.2 (0.4) versus 2.2 (0.1). Chronic conditions [8, 19–21] were also similar between the two groups, with the exception of COPD and Alzheimer’s disease, which were more prevalent in the injurious fall group (see Table 1 below).

**Table 1:** Characteristics of the study population (NAMCS 2016, for visits with patients age 65 years and older)

**Characteristics**	**Total** Value (%)	**Injurious Fall** Value (%)	**Not a Fall** Value (%)	***P-*Value**
**Sample Size, *n***	239,016,482	5,095,734 (2.13)	233,920,748 (97.87)	
**Age Mean (SE)**	74.77 (0.17)	75.28 (1.00)	74.76 (0.17)	0.797
**65–74 years %**	55.29	53.45	55.33	
**75 years and over %**	44.71	46.55	44.68	
**Female %**	55.90	59.01	55.83	0.673
**Race/Ethnicity %**				–
Non-Hispanic White	78.12	87.59	77.91	
Non-Hispanic Black	6.92	6.39	6.94	
Hispanic	10.79	3.91	10.90	
Non-Hispanic Other	4.16	0.00	4.26	
**Chronic Conditions %**				
Cerebrovascular Disease	4.83	6.58	4.79	0.623
Chronic Kidney Disease	6.24	6.97	6.22	0.828
Arthritis	22.33	26.73	22.24	0.513
Depression	9.48	13.17	9.40	0.355
Diabetes Mellitus	22.54	26.55	22.46	0.519
Coronary Artery Disease	15.10	11.91	15.17	0.509
Asthma	45.11	4.23	5.13	0.697
COPD	7.83	18.05	7.61	**0.008**
Alzheimer’s Disease	2.01	6.67	1.91	**0.019**
Osteoporosis	6.48	8.32	6.44	0.591
Obesity	7.57	10.35	17.51	0.447
**Total Number of Chronic Conditions Mean (SE)**	2.16 (0.09)	2.22 (0.37)	2.16 (0.09)	0.092

### Medication use

Patients experiencing an injurious fall had a non-statistically significant higher mean (SE) number of continued medications (defined as medications in their regimens prior to the visit) than those without falls (5.9 (0.9) versus 4.4 (0.4); *p* = 0.096). Most (56.8% [95% CI 46.4%, 67.2%]) patients in the injurious fall group had at least one continued FRID as part of their prescription regimen, versus 49.9% [95% CI 44.9%, 54.8%] of patients in the non-fall group. A larger percentage of patients in the injurious fall group experienced polypharmacy (50.2% versus 39.4%; *p* = 0.145) and poly-FRID (44.9% versus 36.8%; *p* = 0.228), though these differences were non-statistically significant. In the injurious fall group, antihypertensives were the most often continued FRID medication class (44.9%), followed by opioids (14.3%), antidepressants (13.7%), anticonvulsants (13.1%), sedative hypnotics (10.3%), benzodiazepines (9.0%), antispasmodics (4.4%), and antipsychotics (0.7%). The frequencies were similar in the non-fall group.

### Prescribing behaviors at the visit

**Fig. 3:**
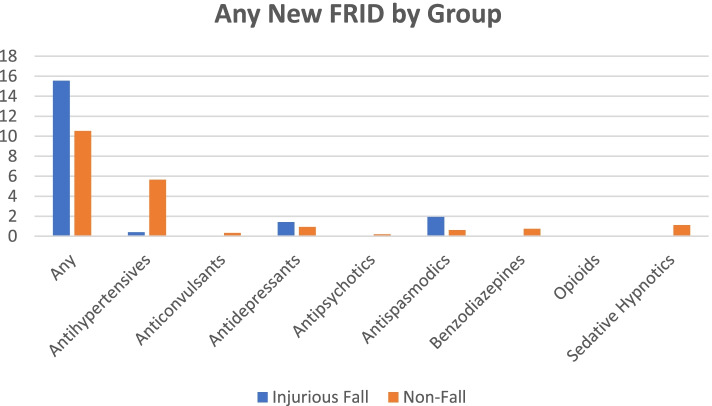
Any New FRID by Group

**Fig. 4:**
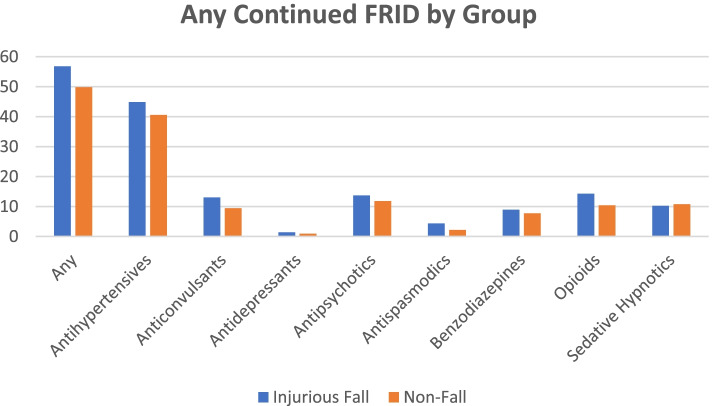
Any Continued FRID by Group

Antihypertensive medications, newly prescribed at the time of the visit, were significantly more prevalent in the non-fall group compared to the injurious fall group (see Table 2 at the end of the text: 5.7% versus 0.4%; *p* < 0.001). Newly prescribed anticonvulsants, antipsychotics, and sedative hypnotics were also more frequent in the non-fall group. In contrast, new antidepressants and antispasmodics were more frequently prescribed in the injurious fall group. Of note, no new anticonvulsants, antipsychotics, benzodiazepines, or opioids were prescribed to the injurious fall group and no new opioids were prescribed to the non-fall group. Patients in the injurious fall group were more likely to be prescribed a new FRID compared to the non-fall group (15.6% [CI 2.3%, 28.8%] versus 10.5% [95% CI 8.9%, 12.2%]), although this difference was modest in magnitude and not statistically significant. See Figs. 3 and 4 for most frequently prescribed (any) new and continued FRID classes by group.

**Table 2:** Prescribing behaviors at the Visit

Variable	Total	Injurious Fall	Not a Fall	*P-*Value
**Number of New Medications Coded Mean (SE)**	0.58 (0.03)	0.41 (0.10)	0.58 (0.03)	0.098
**Number of Continued Medications Coded Mean (SE)**	4.40 (0.26)	5.86 (0.89)	4.36 (0.27)	0.096
**Polypharmacy 5 + continued medications %**	39.60	50.20	39.37	0.145
**Any New FRID %**	10.64	15.55	10.53	0.387
**Total New FRID Mean (SE)**	0.13 (0.01)	0.19 (0.10)	0.13 (0.01) 0	0.553
**Any Continued FRID %**	50.00	56.82	49.85	0.292
**Total Continued FRID Mean (SE)**	1.45 (0.09)	1.79 (0.28)	1.44 (0.09)	0.218
**Poly-FRID 2 + continued FRIDs %**	36.99	44.89	36.82	0.228
**New Antihypertensives %**	5.54	0.40	5.65	** < 0.001**
**Continued Antihypertensives %**	40.70	44.87	40.61	0.524
**New Anticonvulsants %**	0.33	0.00	0.33	–
**Continued Anticonvulsants %**	9.55	13.05	9.47	0.322
**New Antidepressants %**	0.05	1.42	0.94	0.603
**Continued Antidepressants %**	11.89	13.69	11.85	0.696
**New Antipsychotics %**	0.19	0.00	0.19	–
**Continued Antipsychotics %**	1.33	0.66	1.34	0.482
**New Antispasmodics %**	0.65	1.93	0.62	0.257
**Continued Antispasmodics %**	2.27	4.36	2.22	0.333
**New Benzodiazepines %**	0.73	0.00	0.74	–
**Continued Benzodiazepines %**	7.81	8.98	7.79	0.694
**New Opioids %**	0.00	0.00	0.00	–
**Continued Opioids %**	10.51	14.31	10.43	0.295
**New Sedative Hypnotics %**	1.10	0.00	1.12	–
**Continued Sedative Hypnotics %**	10.80	10.25	10.81	0.874

In the logistic regression model adjusted for age at visit, polypharmacy, and presence of chronic conditions (cerebrovascular disease, CKD, arthritis, depression, diabetes, coronary artery disease, asthma, COPD, Alzheimer’s disease, osteoporosis, and obesity), an injurious fall was associated with 1.6 times increased odds (95% CI 0.6, 4.0; *p* = 0.3058 and Bayes factor upper bound = 1.0153) of being prescribed at least one new FRID. See Table 3 below.

**Table 3:** Association between injurious fall and risk of new FRID

Model	Point Estimate	95% Confidence Limits		*P*-Value	Bayes factor upper bound
**Unadjusted**	1.564	0.563	4.341	0.3898	1.0017
**Adjusted model using minimally sufficient set**	1.609	0.647	4.003	0.3058	1.0153
**Adjusted model with age as an effect modifier**					
65–74 years	2.621	0.929	7.399	0.0687	1.9996
> / = 75 years	0.451	0.126	1.620	0.2220	1.1010

The relation of an injurious fall with the odds of being prescribed any new FRID differed by age, though these differences were non-statistically significant. For adults aged 65 to 74 years, an injurious fall was associated with a non-statistically significant 2.6-fold increase in odds (95% CI 0.9, 7.4; *p* = 0.0687 and Bayes factor upper bound = 1.9996) of being prescribed at least one new FRID. For patients aged 75 years of age or older, an injurious fall was associated with non-statistically significant decreased odds, OR 0.40 (95% CI 0.1, 1.6; *p* = 0.2220 and Bayes factor upper bound = 1.1010) of being prescribed at least one new FRID. See Table 3 below.

## Discussion

In this cross-sectional analysis of a nationally representative sample of ambulatory care visits occurring in the United States among adults age 65 years or older, we observed that an injurious fall showed a non-statistically significant increase in odds of being prescribed any new FRID, but these results differed by age. Patients age 65 to 74 years presenting with an injurious fall were more likely to be prescribed any new FRID, in comparison to patients age 75 years or older, who were less likely to be prescribed any new FRID. Though the results were not statistically significant based on *p-*values, the Bayes factor upper bound of 1.9996 for adults 65–74 years indicates the alternative hypothesis (meaning there is a difference in likelihood of being prescribed a new FRID between the two groups) is at most twice as likely as the null hypothesis. In addition, the trends in data suggest possible clinical significance. One might reason that an ambulatory care physician’s level of concern is increased in a patient 75 years or older (as opposed to a younger patient 65–74 years) after an injurious fall. It is possible that increasing frailty often seen with advancing age may lead the provider to place greater weight on the risk of a subsequent fall as opposed to the potential benefits of a newly prescribed FRID (such as better blood pressure control with a new antihypertensive medication) in this higher age group. In contrast, providers may be more willing to risk a subsequent fall in the group of younger patients (age 65–74 years) given increased life expectancy and the potential to improve chronic conditions and associated long-term health outcomes.

Out of the entire sample (all visits with patients 65 years or older), new antidepressants and new antispasmodics were the most frequently prescribed FRID medication classes at the visits occurring due to injurious falls. Patients frequently experienced polypharmacy, which was defined as 5 or more continued medications. Poly-FRID, which was defined as 2 or more continued FRID medications, was also frequently experienced by all patients regardless of fall group.

The results from this cross-sectional analysis have concerning implications for health outcomes in older adults, especially considering the frequent prescribing of new antidepressants and antispasmodics, as well as the widespread issue of polypharmacy across both groups. A meta-analysis found that use of antidepressants in older adults was associated with a 1.4 times increased risk of falls after adjusting for potential confounders [12]. Antispasmodics are also widely understood to increase fall risk, especially in elderly patients, due to their depressive effects (dizziness, drowsiness, and hypotension) on the central nervous system [26]. Antihypertensives, anticonvulsants, antipsychotics, antispasmodics, benzodiazepines, opioids, and sedative hypnotics have also been shown to increase fall risk and should therefore be avoided when possible, especially in patients acutely experiencing an injurious fall [11–13]. Lastly, a meta-analysis of extant studies suggests that polypharmacy is associated with an increased odds of falling (pooled OR 1.75 [1.27, 2.41]) [13]. In addition, the results from this cross-sectional analysis are similar to a recent systematic review that concluded, with limited evidence, that there is a high prevalence of FRID use among older adults who have experienced a fall-related injury and no reduction in overall FRID use following the fall-related healthcare encounter [27].

While the data suggest concerning implications in some realms, there are also positive trends to be noted. First, opioid prescribing was not higher in the injurious group (which might be anticipated given potential pain from the injuries), suggesting that providers may be considering subsequent fall risk in their prescribing practices. In addition, the significant increase in prescription of antihypertensives in the non-fall group likely reflects appropriate ambulatory care prescribing practices (i.e., management of hypertension and prevention of negative cardiovascular health outcomes); the lower frequency in the injurious fall group suggests providers may be balancing the risk of a subsequent fall with the potential benefit of managing chronic conditions.

Pharmacists are uniquely positioned to implement falls-prevention interventions in the older adult population. As one of the most accessible healthcare providers, pharmacists can 1) assist with comprehensive medication management (CMM); 2) provide patients with appropriate education regarding medications that increase fall risk; and 3) counsel patients on non-pharmacologic tips to decrease fall risk.

The CMM is perhaps one of the most effective ways for pharmacists to serve as a liaison between patients and their other health care providers. It is defined as “a patient centered approach to optimizing medication use and improving patient health outcomes that is delivered by a clinical pharmacist working in collaboration with the patient and other health care providers.” [28] During this process, the pharmacist evaluates each of the patient’s medications (including prescription, over-the-counter, and vitamins/herbal supplements) for appropriateness, safety, and efficacy. When the pharmacist identifies a potential issue, they work with the prescriber(s) to either resolve or mitigate the risk.

CMM has been shown to improve patient experience, provide better care, reduce cost, and to improve the provider experience in a variety of settings [28]. One example of when CMM could be effective in preventing falls is when a patient experiences polypharmacy (or poly-FRID). The pharmacist would first raise awareness to both the patient and prescriber(s) about risks associated with polypharmacy (or poly-FRID), and then make recommendations as to which potentially inappropriate medications (PIMs) should be prioritized for deprescribing efforts. In fact, one study showed that withdrawal of fall-risk-increasing drugs is effective as a single intervention for fall prevention [29]. In addition, CMM could be effective in preventing recurrent falls. In outpatient clinic settings, pharmacists included on interprofessional teams can discourage new FRIDs prescriptions, especially at visits related to injurious falls.

Unfortunately, deprescribing FRIDs is not always a viable option, as the disease state being treated often poses more risk than an injury from a hypothetical fall or there are no reasonable medication alternatives. In this case, patient counseling is essential. During any counseling session with a patient age 65 years or older, the pharmacist should first and foremost ask the patient about his/her history of falls. The pharmacist should then discuss medications that are known to independently increase fall risk. One example of a counseling tip for a new or continued antihypertensive medication would be, “Because this medication lowers your blood pressure, you may feel dizzy and lose your balance when standing or sitting up quickly. Be sure to rise slowly.”

Providing such pharmacologic tips to patients is not the only way to decrease fall risk, however. Pharmacists can also suggest simple, non-medication related tips to prevent falls, such as being physically active and wearing sensible shoes. Pharmacists can also educate patients on modifications to their physical residence – illuminating stairs, removing throw rugs on the floor, and placing a nightlight in the path from the bedroom to the bathroom. Lastly, pharmacists can assist patients in selecting durable medical equipment (DME), like canes or walkers, when appropriate.

### Strengths

One strength of this study was the ability to leverage a large, nationally representative dataset to provide estimates of FRID associated with injurious falls. The majority of analyses currently available in the literature include much smaller sample sizes, and the results are not always generalizable to the target population. Another strength of this study was the use of the comprehensive Lexicon Plus® database and the Multum classification system in the classification of FRIDs.

### Limitations

Perhaps the major limitation of this study was the low weighted frequency of injurious falls reported in the NAMCS. Further, we note that our results underestimate the true prevalence of injurious falls for several reasons. First, fall injuries are frequently underreported in older adults, with one study concluding that 72% of individuals who received Medicare-reimbursed health care for fall-related injuries failed to report a fall injury when asked [30]. Second, patients with more severe injuries likely sought care at an emergency department, rather than with their primary care provider, and therefore were not captured in this survey. Third, the NAMCS does not include a section for physicians to record whether patients have experienced a fall-related injury within the recent past, and therefore only acute injuries were likely to be recorded. Despite the low percentage of injurious falls, however, significant knowledge can be gleaned from the results of this study, which highlight the pharmacist’s role in fall prevention.

Another limitation was the cross-sectional nature of the analysis, which ruled out the possibility for any longitudinal follow-up and for causal inference. We were further limited in the inability to observe from the NAMCS data discontinued medications or fall history. Lastly, because sampling of visits per physician was not based on age, we may have introduced selection bias by excluding visits for persons less than 65 years of age.

## Conclusions

Although our effect estimates were not statistically significant, we observed that adults age 65 to 74 years presenting to their ambulatory care visit for an injurious fall showed a non-statistically significant increase in odds of being prescribed a new FRID than their counterparts. In contrast, adults age 75 years or older showed a non-statistically significant decrease in odds of being prescribed a new FRID than their counterparts. Pharmacists can play an important role in preventing recurrent and first-time falls for all older adults, regardless of age, through CMM and patient counseling (both pharmacologic and non-pharmacologic). More studies are needed to further evaluate prescribing behaviors after a fall.

## Supplementary Information

**Additional file 1.** 

## Data Availability

The datasets analyzed during the current study are available publicly on the Centers for Disease Control and Prevention NAMCS/NHAMCS – Ambulatory Health Care Data Homepage (https://www.cdc.gov/nchs/ahcd/about_ahcd.htm).

## Abbreviations

AGS: American Geriatrics Society
DVT: Deep venous thrombosis
CDC: Centers for Disease Control and Prevention
CI: Confidence interval
CKD: Chronic kidney disease
CMM: Comprehensive medication management
COPD: Chronic obstructive pulmonary disease
DME: Durable medical equipment
FRID(s): Fall-risk-increasing-drug(s)
IQR: Interquartile range
NAMCS: National Ambulatory Medical Care Survey
PIMs: Potentially inappropriate medications
RFV: Reason for visit
SE: Standard error
STEADI-Rx: Stopping Elderly Accidents, Deaths, and Injuries
TBI: Traumatic brain injury
