# Supplementary material for: An evaluation of injurious falls and Fall-Risk-Increasing-Drug (FRID) prescribing in ambulatory care in older adults
Source: BMC Geriatr. 2022 Mar 10;22:190. doi: 10.1186/s12877-022-02877-z (PMC8908684; doi:10.1186/s12877-022-02877-z)
Supplement: Supplementary file 1 — Additional file 1. [file 12877_2022_2877_MOESM1_ESM.docx]

**Appendix 1:**

**CAUSES OF INJURY (VARIABLES = CAUSE1, CAUSE2, and CAUSE3)**

'W000' = 'Fall on same level due to ice and snow'

'W100' = 'Fall (on)(from) escalator'

'V800' = 'Animl-rider/occ injured fall from same w/o collision'

'W010' = 'Fall on same level from slip/trip w/o strike against object'

'V920' = 'Drowning and submersion due to fall off watercraft'

'W130' = 'Fall from, out of or through balcony'

'V940' = 'Hitting obj/botm of body of water due to fall from wtrcrft'

'W050' = 'Fall from non-moving wheelchair'

'W160' = 'Fall into swimming pool'

'W170' = 'Fall into well'

'W180' = 'Fall due to bumping against object'

'W090' = 'Fall on or from playground slide'

'W001' = 'Fall from stairs and steps due to ice and snow'

'W101' = 'Fall (on)(from) sidewalk curb'

'W011' = 'Fall on same level from slip/trip w strike against object'

'Y211' = 'Drown after fall into bathtub, undetermined intent'

'W131' = 'Fall from, out of or through bridge'

'W051' = 'Fall from non-moving nonmotorized scooter'

'W161' = 'Fall into natural body of water'

'W171' = 'Fall into storm drain or manhole'

'W181' = 'Fall from or off toilet'

'W091' = 'Fall from playground swing'

'W002' = 'Other fall from one level to another due to ice and snow'

'W102' = 'Fall (on)(from) incline'

'V902' = 'Drown due to falling or jumping from burning watercraft'

'V912' = 'Fall due to collision betw watercraft and oth wtrcrft/obj'

'W132' = 'Fall from, out of or through roof'

'W052' = 'Fall from non-moving motorized mobility scooter'

'W162' = 'Fall in (into) filled bathtub or bucket of water'

'W172' = 'Fall into hole'

'W182' = 'Fall in (into) shower or empty bathtub'

'W092' = 'Fall on or from jungle gym'

'X003' = 'Fall from burning building or structure in uncontrolled fire'

'V903' = 'Drown due to falling or jumping from crushed watercraft'

'X013' = 'Fall due to uncontrolled fire, not in building or structure'

'Y213' = 'Drown after fall into swimming pool, undetermined intent'

'X023' = 'Fall from burning building or structure in controlled fire'

'X033' = 'Fall due to controlled fire, not in building or structure'

'W133' = 'Fall through floor'

'V933' = 'Fall on board watercraft'

'W163' = 'Fall into other water'

'W173' = 'Fall into empty swimming pool'

'W183' = 'Other and unspecified fall on same level'

'W134' = 'Fall from, out of or through window'

'W164' = 'Fall into unspecified water'

'W174' = 'Fall from dock'

'W184' = 'Slipping, tripping and stumbling without falling'

'W184' = 'Slipping, tripping and stumbling without falling'

'V815' = 'Occupant of rail trn/veh injured by fall in rail trn/veh'

'V825' = 'Occupant of streetcar injured by fall in streetcar'

'V816' = 'Occupant of rail trn/veh injured by fall from rail trn/veh'

'V826' = 'Occupant of streetcar injured by fall from streetcar'

'W108' = 'Fall (on) (from) other stairs and steps'

'W138' = 'Fall from, out of or through other building or structure'

'W178' = 'Other fall from one level to another'

'W098' = 'Fall on or from other playground equipment'

'W009' = 'Unspecified fall due to ice and snow'

'W109' = 'Fall (on) (from) unspecified stairs and steps'

'W139' = 'Fall from, out of or through building, not otherwise spcf'

'Y30X' = 'Falling, jumping or pushed from a high place, undetermined intent'

'W12X' = 'Fall (on) (from) scaffolding'

'W03X' = 'Other fall on same level due to collision with another person'

'W04X' = 'Fall while being carried or supported by other persons'

'W14X' = 'Fall from tree'

'W15X' = 'Fall from cliff'

'W06X' = 'Fall from bed'

'W07X' = 'Fall from chair'

'W08X' = 'Fall from other furniture'

'W19X' = 'Unspecified fall'

**REASONS FOR VISIT (VARIABLES = RFV1, RFV2, RFV3, RFV4, RFV5)**

***Only considered for injurious fall if causes of injury variables were blank**

50100 = 'Fracture/dislocation of spinal column'

50150 = 'Fracture/dislocation of trunk area, e...'

50200 = 'Fracture/dislocation of leg'

50250 = 'Fracture/dislocation of ankle'

50300 = 'Fracture/dislocation of foot and toes'

50350 = 'Fracture/dislocation of arm'

50400 = 'Fracture/dislocation of wrist'

50450 = 'Fracture/dislocation of hand and fingers'

50500 = 'Fracture, other and unspecified'

51050 = 'Sprain and strain of cervical spine, ...'

51100 = 'Sprain and strain of back'

51150 = 'Sprain and strain of knee'

51200 = 'Sprain and strain of ankle'

51250 = 'Sprain and strain of wrist'

51300 = 'Sprain or strain, other and unspecified'

54050 = 'Contusions/abrasions/bruises of head,...'

54100 = 'Contusions/abrasions/bruises of eye'

54150 = 'Contusions/abrasions/bruises of trunk...'

54200 = 'Contusions/abrasions/bruises of lower...'

54250 = 'Contusions/abrasions/bruises of upper...'

54300 = 'Contusion/abrasion/bruise, site unspe...'

55050 = 'Injury, other and unspecified of head...'

55100 = 'Injury, other and unspecified, of eye'

55150 = 'Injury, other and unspecified, of back'

55200 = 'Injury, other and unspecified, of che...'

55250 = 'Injury, other and unspecified, of hip'

55300 = 'Injury, other and unspecified, of leg'

55350 = 'Injury, other and unspecified, of knee'

55400 = 'Injury, other and unspecified, of ankle'

55450 = 'Injury, other and unspecified, of foo...'

55500 = 'Injury, other and unspecified, of sho...'

55550 = 'Injury, other and unspecified, of arm'

55600 = 'Injury, other and unspecified, of elbow'

55650 = 'Injury, other and unspecified, of wrist'

55700 = 'Injury, other and unspecified, of han...'

55750 = 'Injury, multiple or unspecified'

**Appendix 2**

**ANTICONVULSANT**

'064'='CNS; Anticonvulsants'

'199'='CNS; Anticonvulsants; Hydantoin anticonvulsants'

'200'='CNS; Anticonvulsants; Succinimide anticonvulsants'

'201'='CNS; Anticonvulsants; Barbiturate anticonvulsants'

'202'='CNS; Anticonvulsants; oxazolidinedione anticonvulsants'

'203'='CNS; Anticonvulsants; Benzodiazepine anticonvulsants'

'204'='CNS; Anticonvulsants; Miscellaneous anticonvulsants'

'311'='CNS; Anticonvulsants; Dibenzapine anticonvulsants'

'345'='CNS; Anticonvulsants; Fatty acid derivate anticonvulsants'

'346'='CNS; Anticonvulsants; Gamma-aminobutyric acid reuptake inhibitors'

'347'='CNS; Anticonvulsants; Gamma-aminobutyric acid analogs'

'348'='CNS; Anticonvulsants; Triazine anticonvulsants'

'349'='CNS; Anticonvulsants; Carbamate anticonvulsants'

'350'='CNS; Anticonvulsants; Pyrrolidine anticonvulsants'

'351'='CNS; Anticonvulsants; Carbonic anhydrase inhibitor anticonvulsants'

'352'='CNS; Anticonvulsants; Urea anticonvulsants'

'446'='CNS; Anticonvulsants; Neuronal potasssium channel openers'

**ANTIDEPRESSANT**

'249'='Psychotherapeutic agents; antidepressants'

'076'='Psychotherapeutic agents; antidepressants; miscellaneous antidepressants'

'208'='Psychotherapeutic agents; antidepressants; SSRI antidepressants'

'209'='Psychotherapeutic agents; antidepressants; tricyclic antidepressants'

'250'='Psychotherapeutic agents; antidepressants; monoamine oxidase inhibitors'

'306'='Psychotherapeutic agents; antidepressants; phenylpiperazine antidepressants'

'307'='Psychotherapeutic agents; antidepressants; tetracyclic antidepressants'

'308'='Psychotherapeutic agents; antidepressants; SSNRI antidepressants'

**ANTIHTN**

'041'='Cardiovascular agents; agents for hypertensive emergencies'

'042'='Cardiovascular agents; angiotensin converting enzyme inhibitors'

'043'='Cardiovascular agents; antiadrenergic agents, peripherally acting'

'044'='Cardiovascular agents; antiadrenergic agents, centrally acting'

'045'='Cardiovascular agents; antianginal agents'

'046'='Cardiovascular agents; antiarrhythmic agents'

'047'='Cardiovascular agents; beta-adrenergic blocking agents'

'274'='Cardiovas agents; beta-adrenergic blocking; cardioselective beta blockers'

'275'='Cardiovas agents; beta-adrenergic blocking; non-cardioselective beta blockers'

'048'='Cardiovascular agents; calcium channel blocking agents'

'049'='Cardiovascular agents; diuretics'

'154'='Cardiovascular agents; diuretics; loop diuretics'

'155'='Cardiovascular agents; diuretics; potassium-sparing diuretics'

'156'='Cardiovascular agents; diuretics; thiazide and thiazide-like diuretics'

'157'='Cardiovascular agents; diuretics; carbonic anhydrase inhibitors'

'158'='Cardiovascular agents; diuretics; miscellaneous diuretics'

'052'='Cardiovascular agents; peripheral vasodilators'

'053'='Cardiovascular agents; vasodilators'

'055'='Cardiovascular agents; antihypertensive combinations'

'056'='Cardiovascular agents; angiotensin II inhibitors'

'303'='Cardiovascular agents; agents for pulmonary hypertension'

'319'='Cardiovascular agents; vasopressin antagonists'

'340'='Cardiovascular agents; aldosterone receptor agonists'

'342'='Cardiovascular agents; renin inhibitors'

'385'='Cardiovascular agents; antiarrhythmic agents; group I antiarrhythmics'

'386'='Cardiovascular agents; antiarrhythmic agents; group II antiarrhythmics'

'387'='Cardiovascular agents; antiarrhythmic agents; group III antiarrhythmics'

'388'='Cardiovascular agents; antiarrhythmic agents; group IV antiarrhythmics'

'389'='Cardiovascular agents; antiarrhythmic agents; group V antiarrhythmics'

'467'='Cardiovascular agents; antihypertensive combinations; ACE inhibitors with thiazides'

'468'='Cardiovascular agents; antihypertensive combinations; Antiadrenergic agents (central) with thiazides'

'469'='Cardiovascular agents; antihypertensive combinations; Antiadrenergic agents (peripheral) with thiazides'

'470'='Cardiovascular agents; antihypertensive combinations; Miscellaneous antihypertensive combinations'

'472'='Cardiovascular agents; antihypertensive combinations; Beta blockers with thiazides'

'473'='Cardiovascular agents; antihypertensive combinations; Angiotensin II inhibitors with thiazides'

'474'='Cardiovascular agents; antihypertensive combinations; Beta blockers with calcium channel blockers'

'475'='Cardiovascular agents; antihypertensive combinations; Potassium sparing diuretics with thiazides'

'476'='Cardiovascular agents; antihypertensive combinations; ACE inhibitors with calcium channel blocking agents'

'479'='Cardiovascular agents; antihypertensive combinations; Angiotensin II inhibitors with calcium channel blockers'

**ANTIPSYCH**

'077'='Psychotherapeutic agents; antipsychotics; miscellaneous antipsychotic agents'

'079'='Psychotherapeutic agents; antipsychotics; psychotherapeutic combinations'

'210'='Psychotherapeutic agents; antipsychotics; phenothiazine antipsychotics'

'280'='Psychotherapeutic agents; antipsychotics; thioxanthenes'

'341'='Psychotherapeutic agents; antipsychotics; atypical antipsychotics'

'251'='Psychotherapeutic agents; antipsychotics'

**ANTISPASM**

'074'='CNS; Muscle relaxants; neuromuscular blocking agents'

'178'='CNS; Muscle relaxants; skeletal muscle relaxants'

'179'='CNS; Muscle relaxants; muscle relaxants combinations'

**BZD**

'069'='CNS; Anxiolytics, sedatives, and hypnotics; benzodiazepines'

'203'='CNS; Anticonvulsants; Benzodiazepine anticonvulsants'

**OPIOID**

'060'='CNS; analgesics; narcotic'

'191'='CNS; analgesics; narcotic analgesic combinations'

**SEDHYP**

'067'='CNS; Anxiolytics, sedatives, and hypnotics'

'068'='CNS; Anxiolytics, sedatives, and hypnotics; barbiturates'

'069'='CNS; Anxiolytics, sedatives, and hypnotics; benzodiazepines'

'070'='CNS; Anxiolytics, sedatives, and hypnotics; miscellaneous'
